# Supplementary material for: Orally Delivered Connexin43 Hemichannel Blocker, Tonabersat, Inhibits Vascular Breakdown and Inflammasome Activation in a Mouse Model of Diabetic Retinopathy
Source: Int J Mol Sci. 2023 Feb 15;24(4):3876. doi: 10.3390/ijms24043876 (PMC9961562; doi:10.3390/ijms24043876)
Supplement: Supplementary file 1 [file ijms-24-03876-s001.zip › ijms-2188774-supplementary.pdf]

Supplementary File

Orally Delivered Connexin43 Hemichannel Blocker, Tonabersat, Inhibits Vascular Breakdown and Inflammasome Activation in a Mouse Model of Diabetic Retinopathy

Odunayo O. Mugisho [1], Jyoti Aryal [1], Avik Shome [1], Heather Lyon [1], Monica L. Acosta [2], Colin R. Green [3,*] and Ilva D. Rupenthal [1]

[1] Buchanan Ocular Therapeutics Unit, Department of Ophthalmology, University of Auckland, Auckland 1023, New Zealand
[2] School of Optometry and Vision Science, University of Auckland, Auckland 1023, New Zealand
[3] Department of Ophthalmology, University of Auckland, Auckland 1023, New Zealand
* Correspondence: c.green@auckland.ac.nz; Tel.: +64-9-923-7599

Online Resource Figure S1

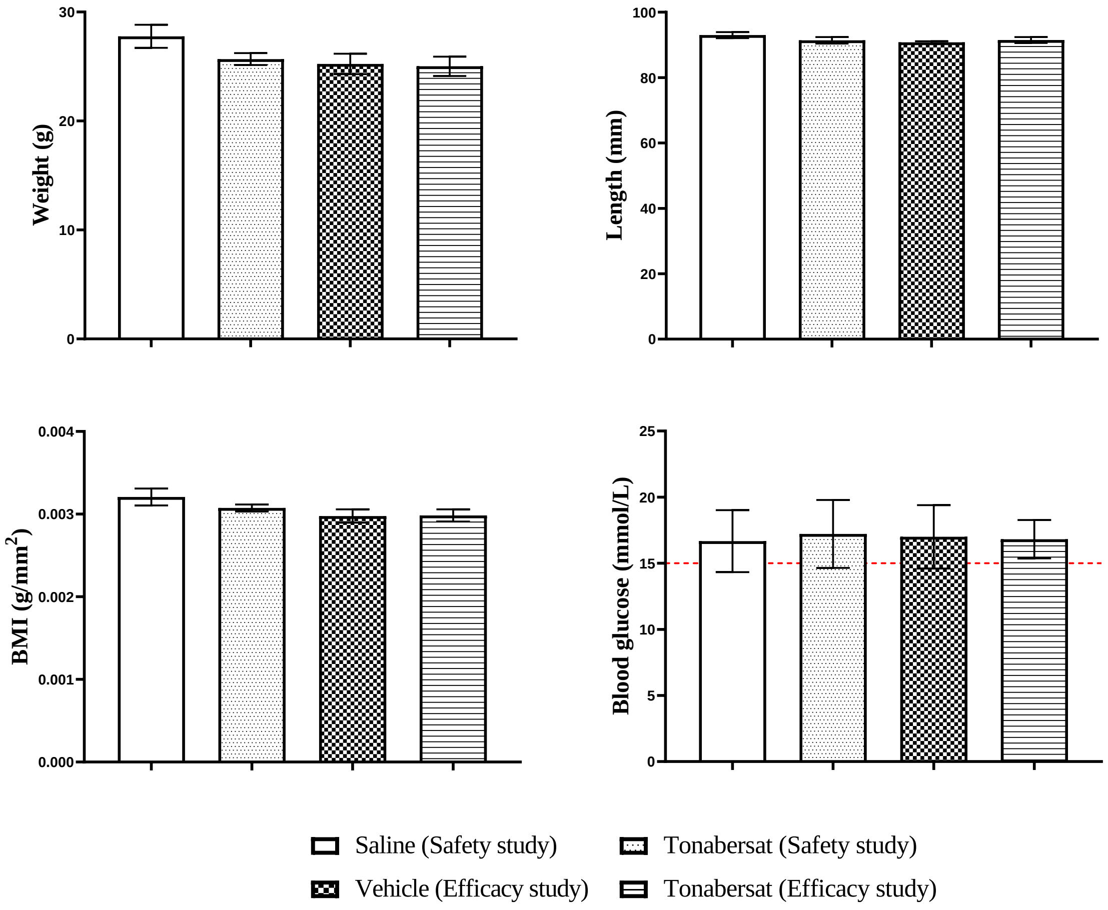

Online Resource Figure S1: There were no statistically significant differences in terms of weight (g), length (mm), BMI (g/mm^2) and blood glucose (mmol/L) between all experimental groups used in this study.

Online Resource Figure S2

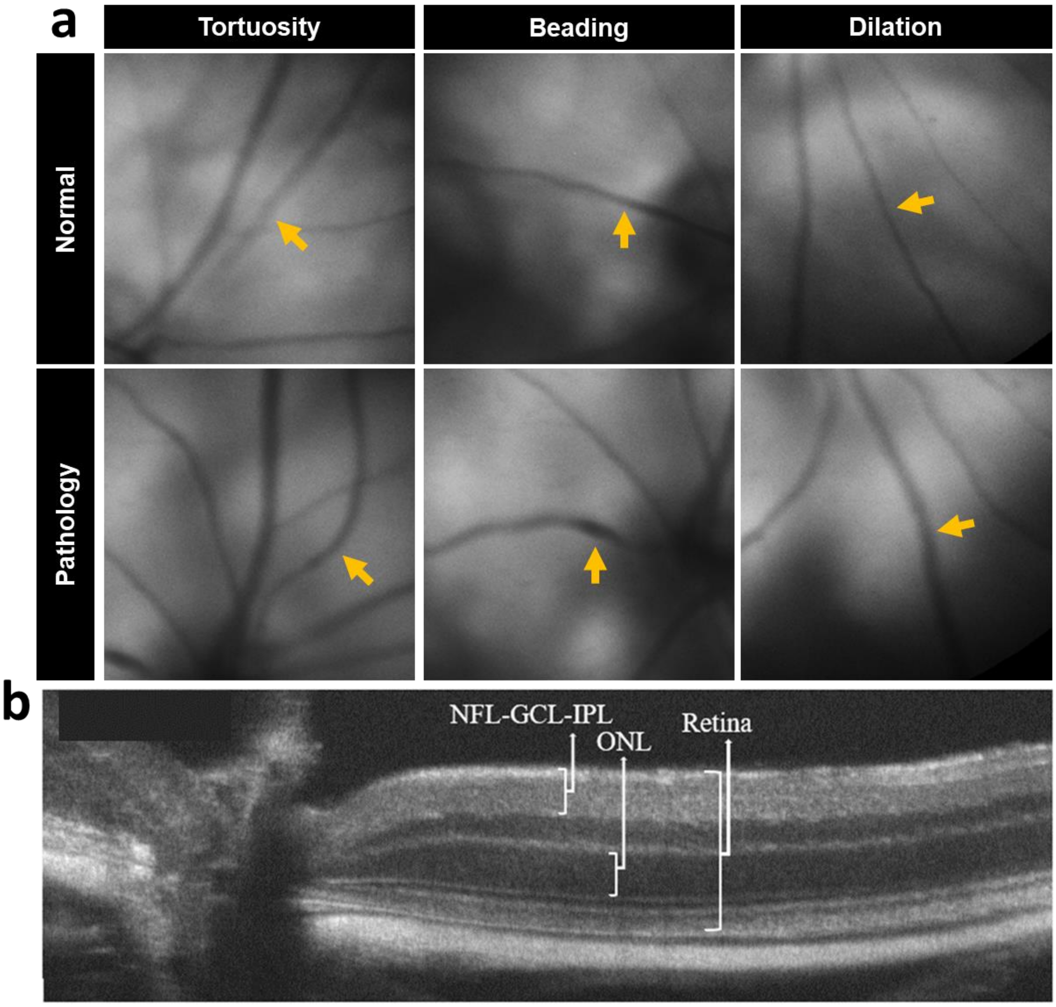

Online Resource Figire S2 (a) Example images highlighting macrovascular changes (vessel tortuosity, beading, and dilation) assessed in this study. Yellow arrows have been used to highlight affected blood vessels. **(b)** A schematic representation of the different retinal layers. NFL = nerve fiber layer; GCL = ganglion cell layer; IPL = inner plexiform layer; ONL = outer nuclear layer.

Methods for Online Resource Figure S3

Cell culture studies

Human adult retinal pigment epithelial cells (ARPE-19; American Type Culture Collection, USA) were cultured in Dulbecco's Modified Eagle Medium Nutrient Mixture F-12 (DMEM-F12; Thermo Fisher Scientific, Auckland, New Zealand) supplemented with 10% fetal bovine serum (FBS; Invitrogen, Auckland, New Zealand) and a 1x antibiotics and antimycotics mixture (AA, 100x stock) at 37 °C in a humidified 5% CO_2 incubator. Cells were grown in T75 flasks, and the medium was changed twice per week until confluent. Cells were split at a density of 2×10^5 cells/mL into 96-well plates for cell viability studies, 24-well plates for cytokine profiling assessments or 8-chamber culture slides for immunohistochemistry. Cells were then treated with tonabersat (MedChemExpress, Princeton, New Jersey, USA) dissolved in 100% dimethyl sulfoxide (DMSO) at 20, 50, or 100 µM and incubated for 24 h. The final concentration of DMSO in media was 0.1% which has previously been shown to not induce any toxicity in these cells.

Cell viability using an MTT assay

Cells were cultured in six replicates per treatment group, then treated with basal media or 20, 50, and 100 µM of tonabersat in media for 24 h. Following incubation, spent media was removed and 100 µL of MTT (3-(4, 5-dimethylthiazolyl-2)-2, 5-diphenyltetrazolium bromide) solution (5 mg/mL) was added to each well and incubated at 37 °C for 4 h. The MTT solution was then removed and replaced with 100 µL of 0.04 M HCl in absolute isopropanol. Absorbance was measured using a SpectraMax i3x spectrophotometer (Molecular Devices, San Jose, CA, USA) at a wavelength of 570 nm with background subtraction at 650 nm.

Cytokine release profiling using a Luminex Bead Assay

A Luminex bead assay (R&D Systems, Magnetic Luminex Assay, Cat No.- LXSAHM, Minneapolis, MN, USA) was used to measure soluble cytokines in cell culture media. Cells were cultured in triplicates in 24-well plates in basal media or supplemented with tonabersat (100 µM). After 24 h, samples of 3 x 50 µL were taken from each well and transferred into a 96-well flat-bottom plate. The assay was conducted according to the manufacturer's instructions. Briefly, a six-point standard curve for each cytokine (IL-1β, TNF-α, IL-6, IL-8, IL-18, and VEGF) was prepared using the cytokine standards provided. Samples and cytokine standards were incubated in magnetic beads conjugated with the primary antibodies corresponding to the cytokines for 2 h. This was followed by a 1 h incubation with a biotin-antibody cocktail and then 30 min with streptavidin-phycoerythrin. Each incubation was done at room temperature on a plate shaker at 800 rpm. The fluorescence intensity of each bead was measured using a Luminex MAGPIX instrument (R&D Systems, USA). The mean fluorescence intensity for each bead cluster was converted into cytokine concentrations based on the six-point standard curve using xPonent™ software (version 4.2, R&D Systems, USA).

Immunocytochemistry

Cells were cultured in quadruplicates in 8-chamber slides and incubated in either basal medium or 100 µM tonabersat for 24 h. Following treatment, cells were fixed with 4% paraformaldehyde for 10 min and then permeabilized for a further 10 min with 0.1% Triton X-100 in PBS. Cells were blocked in normal goat serum for 1 h and then incubated overnight at 4 °C with rabbit anti-NF-κB (1:1000; Abcam, UK). Following two 10 min washes, cells were incubated at room temperature for 2 h with goat anti-rabbit Alexa-488 (1:500; Abcam, UK). Cell nuclei were stained with DAPI (1:1000; Sigma-Aldrich, USA), then slides were mounted using Citifluor™ anti-fade reagent (Citifluor™, Hatfield, PA, USA) and sealed with nail polish. Fluorescence confocal images were taken on an Olympus FV1000 confocal laser scanning microscope (Olympus, Tokyo, Japan), and processed using Olympus FV10-ASW viewer. Five images were taken from each chamber with experiments repeated three times.

Online Resource Figure S3

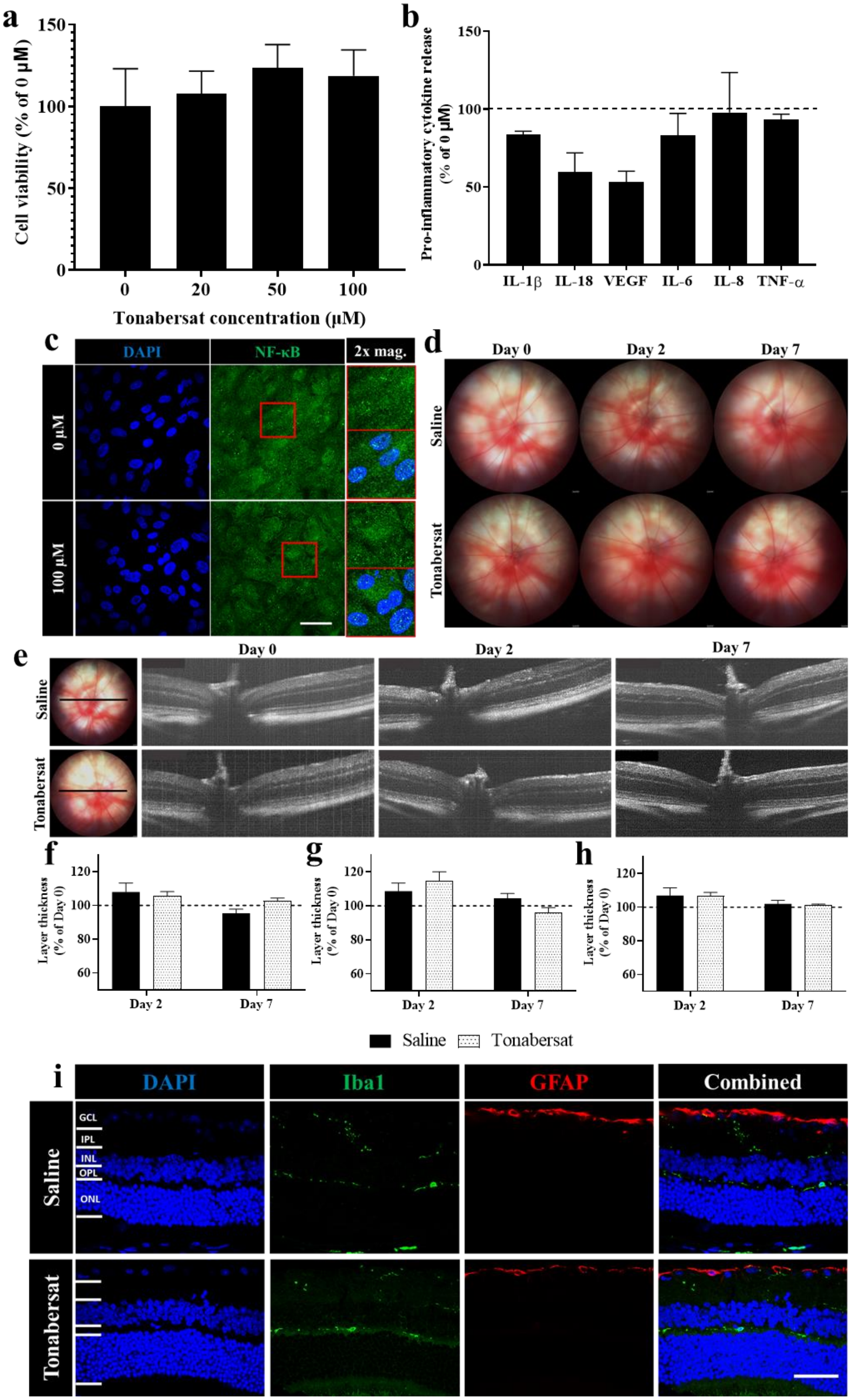

Online Resource Figure S3 Blocking connexin43 hemichannels with tonabersat did not cause any changes under physiological conditions in ARPE-19 cells or NOD mice. **(a)** Tonabersat (20, 50, and 100 µM) did not affect ARPE-19 cell viability relative to basal conditions. **(b)** Tonabersat (100 µM) decreased IL-1β, IL-18, and VEGF but not IL-6, IL-8 and TNF-α release relative to basal conditions (0 µM). **(c)** Tonabersat (100 µM) did not induce NF-κB (green) translocation into cell nuclei (stained with DAPI). Statistical analyses were carried out using one-way ANOVA with Dunnett's multiple comparisons test. n = 6-7 per group. Scale bar = 50 µm. **(d)** Tonabersat treatment did not affect retinal macrovasculature in fundus images. **(e)** OCT images showed that there were no intraretinal abnormalities **(f-h)** There were no significant changes in the thickness of the **(f)** NFL, GCL and IPL, **(g)** ONL or **(h)** overall retina with tonabersat treatment relative to the vehicle group. Statistical analyses were carried out using a two-way ANOVA with Dunnett's multiple comparisons test. **(i)** Tonabersat treatment did not induce GFAP (red) upregulation or Iba1-positive (green) cell activation relative to the vehicle group. GFAP expression was restricted to the GCL and Iba1-positive cells were restricted to the plexiform layers with no Iba-1-positive processes observed within the ONL in both treatment groups. Cell nuclei were stained with DAPI. GCL = ganglion cell layer; IPL = inner plexiform layer; INL = inner nuclear layer; OPL = outer plexiform layer; ONL = outer nuclear layer. Scale bar = 50 µm. n = 6-7 eyes per group

Online Resource Figure S4

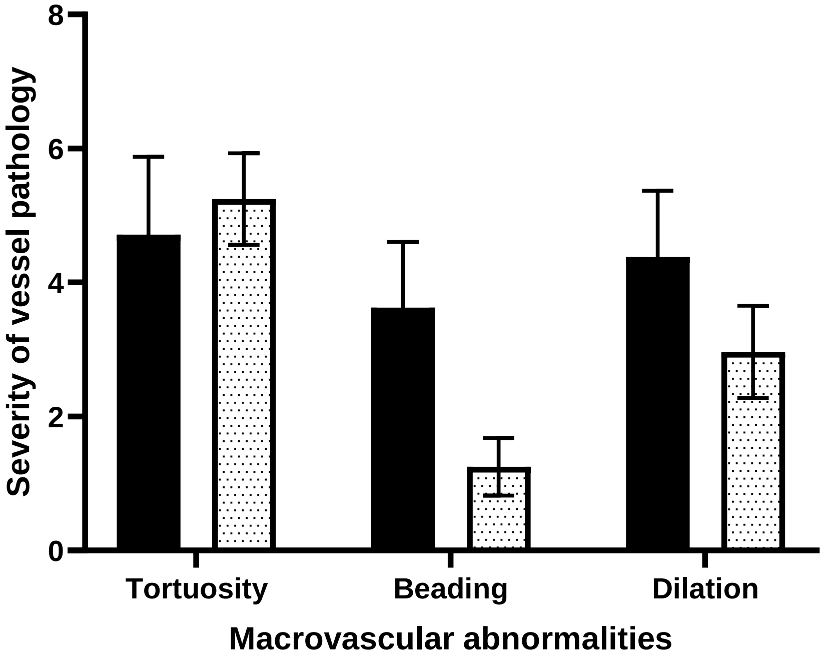

Online Resource Figure S4 Tonabersat pre-treatment does not affect the severity of macrovascular abnormalities (tortuosity, beading and dilation. Data are presented as an mean + standard error of the mean (SEM). Statistical analyses were carried out using two-way ANOVA with Sidak's multiple comparisons test. n = 8 – 12 eyes per group.
